# Keeping CDK18 in balance to prevent DNA replication stress in breast cancer

**DOI:** 10.18632/oncotarget.26517

**Published:** 2018-12-28

**Authors:** Esmée Braams, Vincenzo D'Angiolella

**Affiliations:** Medical Research Council Institute for Radiation Oncology, Department of Oncology, University of Oxford, Oxford, UK

**Keywords:** CDKs, CDK18, breast cancer, cyclins, DNA replication stress

Cyclin Dependent Kinases (CDKs) are serine/threonine kinases, which promote cell cycle progression. The general rule is that CDKs use a cyclin protein to become active. The oscillation of cyclins attached to their respective CDK partners drive the transition from one cell cycle phase to the next. Thus, CDK4/6 partner with D-type cyclins to drive cell cycle entry, CDK2 is activated by cyclin E to promote S phase progression and DNA synthesis, while A and B type cyclins bind to CDK1 to promote the transition from S phase to mitosis (reviewed in [1]). In addition to the aforementioned canonical CDKs which work with a cyclin pair, multiple other CDKs and cyclins exist which are not major cell cycle drivers, however they fine tune important cell cycle events to ensure proper checkpoint control, and prevent mistakes occurring during DNA replication. One example is constituted by cyclin F, which does not partner with a CDK, but instead forms a multi-subunit E3 ubiquitin ligase to promote the ubiquitylation and consequent degradation of specific proteins [2]. Another important example is constituted by CDK18, whose role was recently identified by studies in Collins laboratory [3]. Further studies in the accompanying manuscript not only identified CDK18 as an important regulator of DNA replication stress signalling, but also provided novel insights on its role in breast cancer progression [4].

CDK18 belongs to the PCTAIRE family of CDKs, which includes CDK16, CDK17 and CDK18. They share in common a PCTAIRE amino acid sequence in the helical α-C region of the kinase N-lobe employed by other CDKs to recruit respective cyclin partners. However, the cyclin partner for CDK18 has remained elusive. It has been suggested that CDK18 can interact with cyclin A2 and cyclin E. CDK18 activity is promoted by interaction with cyclin A2 for the phosphorylation of Retinoblastoma protein [5]. More studies are required to establish the mode of action of CDK18 and most importantly to understand the functional role of CDK18. From this point of view, recent studies from the Collis laboratory has identified CDK18 as an important novel regulator of genome stability. Depletion of CDK18 increased endogenous DNA damage and chromosomal abnormalities and in response to replication stress CDK18 promoted the activation of ATR-mediated signalling [3]. CDK18, therefore, represents a novel anti-cancer drug target, however more studies are necessary to clarify the role of CDK18 in cancer cell survival.

G. Barone et al. observed that in breast cancer cohorts, CDK18 gene was amplified. The METABRIC dataset, containing the largest CDK18 gene amplification, showed that increased CDK18 mRNA levels were associated with reduced survival in ER-, but not in ER+ breast cancer. Breast cancers with increased CDK18 mRNA was associated with poor response to chemotherapeutic agents, inducing DNA replication stress. The association was different when the authors analysed CDK18 protein expression in the Nottingham Tenovus breast cancer cohort. In this case, low CDK18 expression was associated with poorer patient survival. High CDK18 protein expression could predict a better response to chemotherapy. These data suggest that CDK18 mRNA and protein levels are regulated differently within cancer cells with potentially distinct predictions on outcome for patients [4].

CDK18 was previously shown to interact with RAD9, a component of the 9-1-1 replication stress signalling complex [3]. This is an important axis to establish an adequate cellular response to replication stress and thus, response to chemotherapy. CDK18 expression could influence the cellular response of tumour cells to chemotherapy. To clarify the role of CDK18 amplification in breast cancer, the authors in the accompanying manuscript increased endogenous CDK18 by using CRISPR activation. After amplification of CDK18, cells were more prone to accumulate DNA damage, visualized by staining with a marker of double strand breaks: γ-H2AX. Importantly, the activation of γ-H2AX was pan nuclear, indicating a diffuse issue with DNA replication. Furthermore, in cells expressing high levels of CDK18, the response to replication stress induced by limiting nucleotides was impaired. The authors observed a compromised activation of ATR signalling and corresponding increased sensitivity to DNA damaging agents impairing nucleotide production such as methotrexate or 5-FU [4].

Overall, the results suggest that elevated levels of CDK18 protein expression likely determined by CDK18 gene amplification observed in breast cancer, confers an improved response to chemotherapeutic agents that induce high levels of replication stress. Therefore, ER- breast cancers with high CDK18 protein levels could benefit from chemotherapeutic agents inducing replication stress.

## References

[1] Lim S (2013). Development.

[2] D'Angiolella V (2013). Trends in Cell Biology.

[3] Barone G (2016). Nucleic Acids Research.

[4] Barone G (2018). Oncotarget.

[5] Matsuda S (2014). J Biol Chem.

